# Gallbladder preserving cholelithotomy in children with hereditary spherocytosis complicated by gallstones: a single-center retrospective study

**DOI:** 10.3389/fped.2024.1457927

**Published:** 2024-11-20

**Authors:** Ran Tang, Cheng-xiao Zhou, Yong Yang, Jian Bian, Li-xiang Meng, De-cheng Wei, Shi-qin Qi

**Affiliations:** Department of Pediatric Surgery, Anhui Provincial Children's Hospital, Hefei, China

**Keywords:** gallbladder-preserving cholelithotomy, pediatric, hereditary spherocytosis, surgery, choledochoscope

## Abstract

**Background:**

Gallstones are among the most common complications of hereditary spherocytosis (HS). In previous treatments, gallbladder-preserving cholelithotomy (GPC) has remained a subject of significant debate due primarily to potential risks of stone recurrence. However, past studies have often overlooked the impact of specific disease conditions on GPC. In this study, we reviewed the clinical data of GPC in HS pediatric patients with concurrent gallstones over a period of seven years in a single center.

**Methods:**

From December 2016 to April 2024, 32 pediatric patients with HS who underwent splenectomy and GPC surgery based on our inclusion criteria. Clinical pathological, and follow-up data of these patients were collected.

**Results:**

In terms of short-term complications, there were there were no cases of postoperative bleeding, bile duct injury. 3 cases (9.3%) experienced varying degrees of bile peritonitis. During long-term follow-up, only 2 cases (6.2%) showed recurrence of gallstones. One case of bile leakage occurred.

**Conclusion:**

GPC demonstrates significant efficacy for pediatric patients with hereditary spherocytosis (HS) complicated by gallstones, showing a a low recurrence rate and high safety profile.

## Introduction

Hereditary spherocytosis (HS) is a familial hemolytic disorder characterized by a defect in the red blood cell membrane, leading to increased red blood cell destruction. In the Chinese population, the incidence rate is 1.27/100,000 for males and 1.49/100,000 for females (1). HS, as a dominant genetic disorder, exhibits familial inheritance and can occur at any age, with a higher prevalence in infants, toddlers, and children, although it is usually definitively diagnosed during childhood or early adulthood (2).

Clinical manifestations of HS include hemolytic anemia, jaundice, and splenomegaly. Prolonged hemolysis can predispose patients to gallstones, and some patients typically present with symptoms of gallstone disease. Splenectomy can significantly improve the clinical symptoms, nearly meeting curative criteria (3).

As cholelithiasis is the most common complication associated with HS, some pediatric HS patients initially present with symptoms such as abdominal pain and fever related to gallstones (4). Along with splenectomy, which is a standard procedure, there is controversy regarding the concurrent management of gallstones. Currently, an increasing number of scholars advocate for gallbladder-preserving cholelithotomy in treating gallstones in children (5). This study aims to retrospectively analyze clinical data from children with HS complicated by gallstones treated at the Children's Hospital of Anhui Province, using laparoscopic combined with choledochoscopy gallbladder-preserving cholelithotomy (GPC) surgery. We analyzed the clinical characteristics of children with HS complicated by gallstones and evaluate the prognosis after simultaneous splenectomy and gallbladder-preserving cholelithotomy to improve the diagnosis and treatment process for HS.

## Methods

We reviewed the clinical data of 32 pediatric patients with moderate to severe HS combined with gallstones admitted to Anhui Children's Hospital from February 2016 to April 17, 2024. Inclusion criteria were: (1) All were moderate to severe HS patients requiring splenectomy (2); (2) Preoperative use of ultrasound and MRI confirmed gallstones; (3) No history of upper abdominal surgery; (4) Gallstone diameter between 1 and 2 cm; (5) Signed informed consent form. Exclusion criteria were: (1) Dodds (6) method used to measure gallbladder contraction function, excluding cases with gallbladder contraction rate ≤30%; (2) Combined common bile duct stones; (3) Patients with concomitant other hematologic diseases were excluded; (4) Cases of gallbladder sludge stones were also excluded, as these patients typically received medical treatment rather than surgery; (5) Cases requiring cholecystectomy due to atrophic gallbladder morphology found during surgery. Patients meeting the inclusion criteria underwent gallbladder-preserving cholecystectomy (GPC) concurrently with splenectomy, and short-term clinical outcomes and long-term stone recurrence rates were evaluated.

A retrospective analysis was conducted on 32 patients who met the inclusion criteria, which included preoperative imaging data such as ultrasound (US) and MRCP examinations, surgical records, and postoperative follow-up data. The postoperative follow-up data included assessments for postoperative acute infections, symptoms of peritonitis (abdominal pain, fever, elevated white blood cell count, and ascites), and recurrence of gallstones.

## Surgical technique

**Figure 1:**
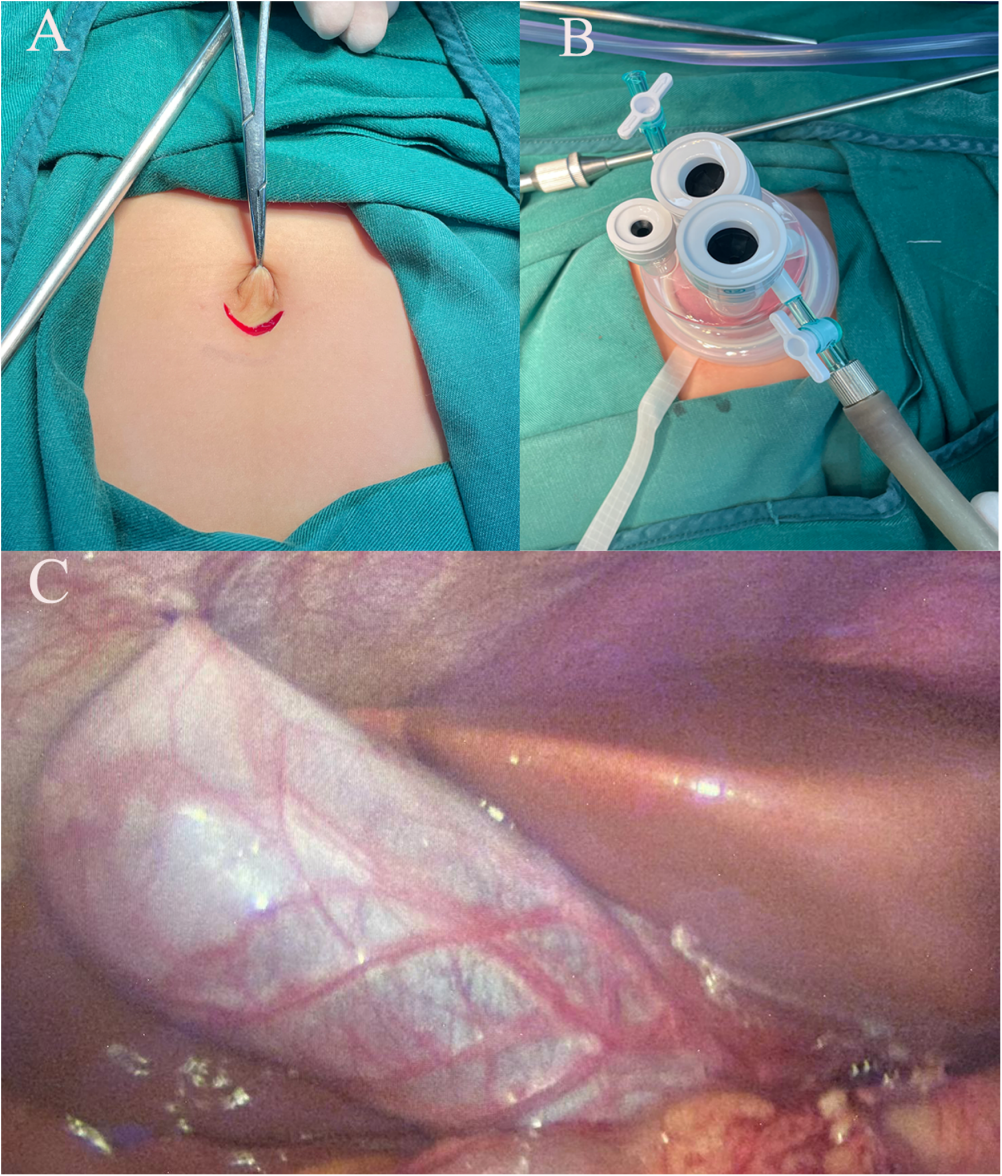
**(A)** A 2−3 cm incision below the belly button; **(B)** Perform the procedure using a single-port laparoscopic access device; **(C)** Suspend the gallbladder using 2-0 silk suture.

**Figure 2:**
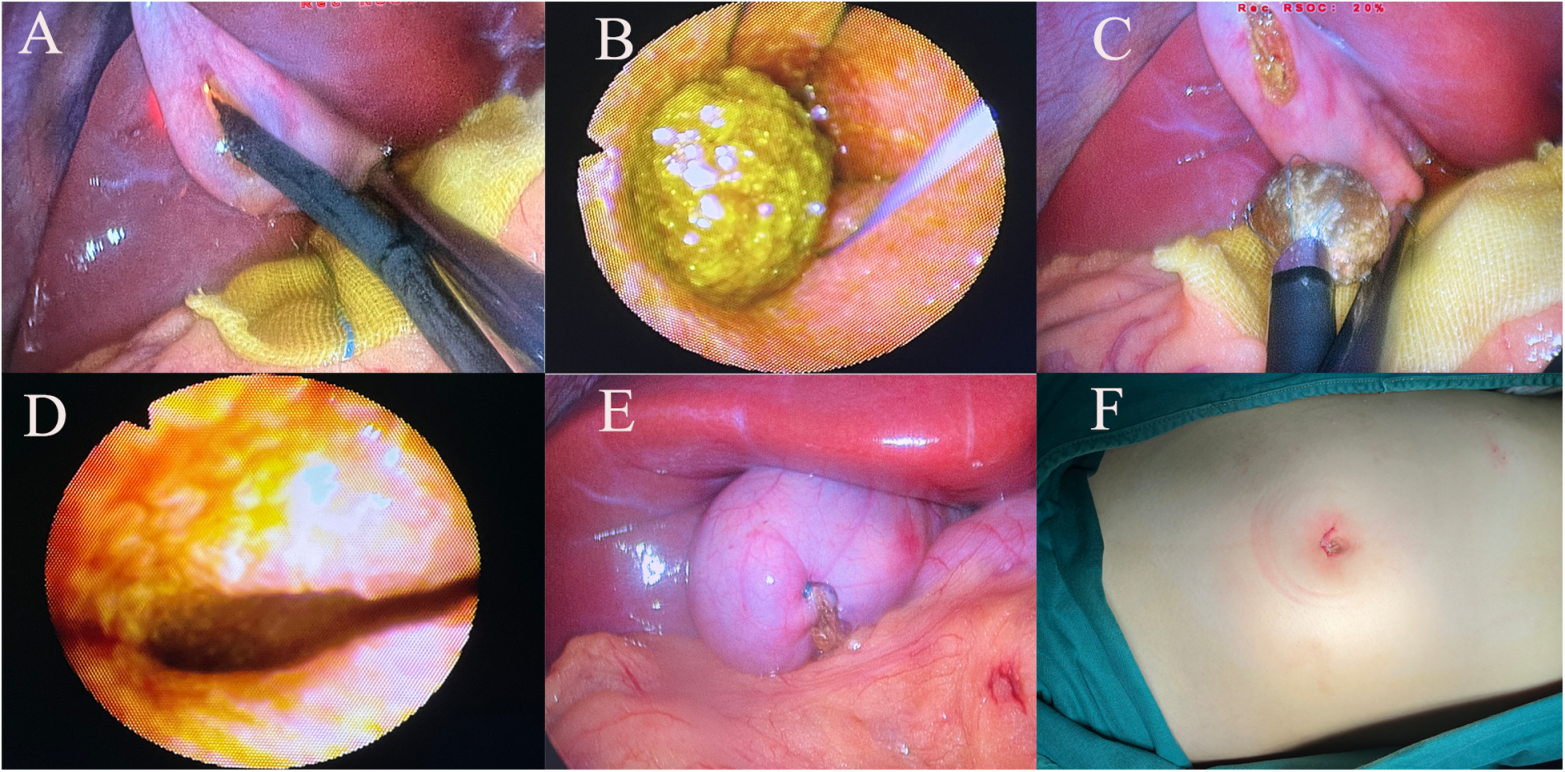
**(A)** Make an approximately 1.5 cm incision in the avascular area at the bottom of the gallbladder, and use a choledochoscopy to explore the gallbladder. **(B,C)** Use a stone retrieval basket to remove the gallstones from the gallbladder; **(D)** The choledochoscope further explores up to the neck of the gallbladder to confirm the absence of residual stones; **(E)** Suture the gallbladder incision with absorbable sutures in a double-layer fashion; **(F)** Suture the umbilical incision with intracutaneous sutures after the surgery.

A 2–3 cm incision is made below the umbilicus (Figure 1A), and a single-port puncture device (Schneider) (Figure 1B) is inserted, establishing pneumoperitoneum with pressure maintained at 8–12 mmHg. After completing the splenectomy, a gallbladder-preserving cholecystolithotomy is performed using choledochoscopy through a single-port. Under laparoscopic guidance, We suspend the gallbladder and observe its morphology (Figure 1C), check for smoothness of its wall, assess for obvious edema or thickening, and evaluate for severe adhesions or significant atrophy to ensure it meets the basic criteria for preservation. Under laparoscopic monitoring, Bulldog Clamps are used to clamp the distal cystic duct to prevent gallstones from entering the common bile duct during gallbladder irrigation. Atraumatic graspers are used to make a 1.5 cm incision in the avascular area of the fundus of the gallbladder after aspirating bile (Figure 2A), followed by insertion of the choledochoscopy to locate and assess the number and size of gallstones.

Using a stone retrieval basket under choledochoscopic visualization, we systematically remove the stones (Figure 2B–C), starting with larger ones in cases of multiple stones and using saline irrigation and suction for smaller fragmented stones, taking care to clamp the cystic duct during irrigation to prevent stone migration into the common bile duct. The choledochoscopy continues until the junction of the cystic duct and common hepatic duct, ensuring no stone residues are left (Figure 2D). Finally, we check for any active bleeding from the gallbladder mucosa, confirm free flow of bile from the cystic duct opening, suture the gallbladder with 4–0 V-Loc (COVIDIEN) (Figure 2E), irrigate the abdominal cavity with saline, confirm the absence of active bleeding or bile leakage, and conclude the surgery. All patients receive routine oral ursodeoxycholic acid therapy postoperatively.

## Results

All 32 patients underwent splenectomy and simultaneous gallbladder-preserving cholecystolithotomy (GPC). Among them, 18 had a single stone, 10 had 2–3 stones, and 4 had 4–8 stones (Table 1). During the postoperative hospitalization period, there were no occurrences of postoperative bleeding, bile duct injury, or worse hepatic insufficiency. Three cases (9.3%) of biliary peritonitis occurred, with one confirmed to be caused by bile leakage. A second surgery was performed to re-suture the gallbladder in this case, while the other two cases recovered after antimicrobial treatment. Upon discharge, they were advised to follow a low-fat diet and take oral ursodeoxycholic acid therapy. All patients were followed up for 1–5 year postoperatively. Two patients (6.2%) experienced stone recurrence, both recurrent patients did not exhibit clinical symptoms, and they are currently under follow-up observation. The remaining patients showed no signs of stone recurrence on follow-up ultrasound and liver function tests, had good gallbladder contraction function, and had no obvious scars on the abdominal incision, with satisfactory outcomes (Table 2).

**Table 1:** Characterization of patients (*n* = 32).

	GPC
Patients (*n*)	32
Mean age (range) (year)	7.5 (5–15)
Sex (*n*)	
Male	17
Female	15
Stone number pre-GPC (*n*)	
Solitary	18
Multiple	14
Clinical presentation, (*n*)	
Abdominal pain	15
Pancreatitis	5
Cholecystitis	12
Asymptomatic	10

GPC, indicates gallbladder-preserving cholelithotomy; The number of stones did not include sandy calculi.

**Table 2:** Operative details and outcomes.

	GPC
Operating time (min)	110 ± 23.4
Type of short-term complication, *n* (%)	
Worse hepatic insufficiency	0
Biliary leakage	1 (3.1%)
Biliary tract injury	0
Biliary peritonitis	3 (9.3%)
Intra-abdominal bleeding	0
Long-term complications, *n* (%)	
Stone recurrence	2 (6.2%)
Recurrent cholecystitis	0

## Discussion

HS is often characterized by recurrent episodes of acute hemolysis within a chronic disease course. Clinical features mainly include anemia, jaundice, and splenomegaly, with a significant importance of family history in HS diagnosis (4). However, due to the atypical nature of HS symptoms and the heterogeneity of clinical manifestations, laboratory tests are often affected, leading to misdiagnosis and underdiagnosis especially in the absence of a specific family history of the disease. Gallstones are one of the common complication of HS, In HS patients under 10 years old, the incidence of gallstones is around 5%–8%. By the second to fifth decades of life, this proportion increases to 40%–50% (7). Most stones form between the ages of 10 and 30. Some HS patients presenting initially with symptoms of acute cholecystitis such as abdominal pain, fever, and jaundice. This is due to the high concentration of bilirubin produced from the destruction of spherocytes in the spleen, which is excreted into the bile, leading to bile stasis and the formation of gallstones in the bile ducts.

For moderate to severe cases of HS, splenectomy is considered the most effective treatment. Splenectomy can almost cure all patients with this disorder, eliminating anemia and hyperbilirubinemia (8, 9). However, there is ongoing debate regarding the optimal management of concurrent gallstones in children with HS undergoing splenectomy. Recent research into gallbladder function has revealed that it is not only an important digestive organ responsible for bile concentration, storage, and regulation of bile duct pressure (10), but also a complex immune organ (11). For gallstone patients with good gallbladder function, gallbladder removal should not be done lightly, as it can lead to a series of unnecessary complications such as bile duct injury, bile reflux gastritis, and hepatoenteric circulation obstructio (12). Especially for HS patients, gallstone formation is caused by the destruction of spherocytes in the spleen. After splenectomy, there is typically no longer a high concentration of bilirubin causing bile stasis. Once the spleen is removed, patients with hereditary spherocytosis will no longer develop pigment stones (13). Therefore, we propose that in HS patients with concurrent gallstones and intact gallbladder function, it is more reasonable to perform GPC along with splenectomy to remove the stones while preserving the gallbladder function.

In our study found that among 32 children with HS and concurrent gallstones who underwent splenectomy and laparoscopic cholecystolithotomy simultaneously, there were there were no cases of postoperative fulminant infection, bile duct injury, or bile duct bleeding. Three cases (9.3%) developed symptoms of bile peritonitis during the postoperative hospitalization period, all of which were eventually cured with antibiotic treatment. Only two cases (6.2%) experienced stone recurrence during the follow-up period after surgery. Compared to traditional laparoscopic cholecystectomy (LC), GPC surgery is safer with fewer complications.

In our analysis, the occurrence of postoperative bile peritonitis was mainly attributed to inadequate irrigation of the abdominal cavity with physiological saline after gallbladder incision during surgery, leading to bile leakage into the abdominal cavity. Additionally, the absence of precise suturing of the gallbladder incision during surgery may also pose a potential risk factor for postoperative bile peritonitis. Since we did not place a drainage tube in the abdominal cavity during our surgeries, precise suturing of the gallbladder incision and thorough irrigation of the abdominal cavity were particularly crucial. With the improvement in surgical team proficiency, the incidence of bile peritonitis can be significantly controlled.

The controversy surrounding GPC for treating gallstones has mainly stemmed from concerns about gallstone recurrence. A single center study indicated that the cumulative 5-year recurrence rate for gallstone patients aged ≤20 was 16.80%, while for patients with single gallstones, the 5-year recurrence rate was 2.87% (5). Previous research (14, 15) suggested that the 5-year recurrence rate after GPC surgery exceeded 30%. Another study (16) reported recurrence rates of 0%, 3.32%, and 5.64% at 0, 36, and 60 months after GPC. Age and the number of gallstones were identified as independent risk factors for gallstone recurrence after GPC under choledochoscopy. However, previous studies lacked strict inclusion criteria for surgical cases, especially regarding the impact of the primary disease on surgical recurrence rates.

HS serves as a distinct pathogenic factor for gallstones. Following splenectomy, there is no longer a high concentration of bilirubin leading to bile stasis. In HS patients, once the spleen is removed, pigment stones do not recur. Chen et al. (17) reported that managing gallbladder stones in HS patients during splenectomy (including 6 cases of cholecystectomy and 8 cases of GPC surgery) can reduce the incidence of adverse events. However, further studies with larger sample sizes are needed to confirm the efficacy of GPC surgery. In our study, among 32 pediatric patients with HS and gallbladder stones who underwent GPC, only 2 experienced recurrence during 1–5 years of follow-up, both of whom had multiple stones preoperatively. Furthermore, postoperative complications were minimal and manageable.

For pediatric patients with HS and concurrent gallstones, a well-functioning gallbladder should not be easily removed, while simple medication therapy often yields unsatisfactory results. Utilizing laparoscopy combined with choledochoscopy for GPC offers advantages such as minimal surgical trauma, fewer postoperative complications, low stone recurrence rates, and aesthetic incisions. This technique is worth exploring due to its benefits. However, it is essential to strictly adhere to surgical indications. For gallstones caused by other diseases, there is still no conclusive evidence proving the efficacy of GPC. Additionally, preoperative and intraoperative assessment of gallbladder function is particularly crucial. Our study has limitations being a single-center study with a limited number of cases meeting the inclusion criteria. In future research, we aim to increase the sample size and strive to conduct multicenter collaborative studies to obtain more convincing data.

## Data Availability

The original contributions presented in the study are included in the article/Supplementary Material, further inquiries can be directed to the corresponding author.

## References

[1] Wang C Cui Y Li Y Liu X Han J. A systematic review of hereditary spherocytosis reported in Chinese biomedical journals from 1978 to 2013 and estimation of the prevalence of the disease using a disease model. Intractable Rare Dis Res. (2015) 4(2):76–81. 10.5582/irdr.2015.01002 25984425 PMC4428190

[2] Bolton-Maggs PH Langer JC Iolascon A Tittensor P King MJ. Guidelines for the diagnosis and management of hereditary spherocytosis–2011 update. Br J Haematol. (2012) 156(1):37–49. 10.1111/j.1365-2141.2011.08921.x 22055020

[3] Perrotta S Gallagher PG Mohandas N. Hereditary spherocytosis. Lancet. (2008) 372(9647):1411–26. 10.1016/S0140-6736(08)61588-3 18940465

[4] Ma S Deng X Liao L Deng Z Qiu Y Wei H Analysis of the causes of the misdiagnosis of hereditary spherocytosis. Oncol Rep. (2018) 40(3):1451–8. 10.3892/or.2018.6578 30015979

[5] Liu J Zhu X Zhao Q Huang K Zhou D Zhang X A new operation for gallstones: choledochoscopic gallbladder-preserving cholecystolithotomy, a retrospective study of 3,511 cases. Surgery. (2022) 172(5):1302–8. 10.1016/j.surg.2022.08.008 36089424

[6] Dodds WJ Groh WJ Darweesh RM Lawson TL Kishk SM Kern MK. Sonographic measurement of gallbladder volume. AJR Am J Roentgenol. (1985) 145(5):1009–11. 10.2214/ajr.145.5.1009 3901703

[7] Tamary H Aviner S Freud E Miskin H Krasnov T Schwarz M High incidence of early cholelithiasis detected by ultrasonography in children and young adults with hereditary spherocytosis. J Pediatr Hematol Oncol. (2003) 25(12):952–4. 10.1097/00043426-200312000-00009 14663278

[8] Eber S Lux SE. Hereditary spherocytosis–defects in proteins that connect the membrane skeleton to the lipid bilayer. Semin Hematol. (2004) 41(2):118–41. 10.1053/j.seminhematol.2004.01.002 15071790

[9] Reliene R Mariani M Zanella A Reinhart WH Ribeiro ML Del GE Splenectomy prolongs *in vivo* survival of erythrocytes differently in spectrin/ankyrin- and band 3-deficient hereditary spherocytosis. Blood. (2002) 100(6):2208–15. 10.1182/blood.V100.6.2208 12200387

[10] Sahu S Joglekar MV Dumbre R Phadnis SM Tosh D Hardikar AA. Islet-like cell clusters occur naturally in human gall bladder and are retained in diabetic conditions. J Cell Mol Med. (2009) 13(5):999–1000. 10.1111/j.1582-4934.2008.00572.x 19175681 PMC3823415

[11] Walker SK Maki AC Cannon RM Foley DS Wilson KM Galganski LA Etiology and incidence of pediatric gallbladder disease. Surgery. (2013) 154(4):927–31; discussion 931–3. 10.1016/j.surg.2013.04.040 24074432

[12] Fisher M Spilias DC Tong LK. Diarrhoea after laparoscopic cholecystectomy: incidence and main determinants. Anz J Surg. (2008) 78(6):482–6. 10.1111/j.1445-2197.2008.04539.x 18522570

[13] Sandler A Winkel G Kimura K Soper R. The role of prophylactic cholecystectomy during splenectomy in children with hereditary spherocytosis. J Pediatr Surg. (1999) 34(7):1077–8. 10.1016/S0022-3468(99)90569-9 10442593

[14] Zou YP Du JD Li WM Xiao YQ Xu HB Zheng F Gallstone recurrence after successful percutaneous cholecystolithotomy: a 10-year follow-up of 439 cases. Hepatobiliary Pancreat Dis Int. (2007) 6(2):199–203. 17374582

[15] De Caluwe D Akl U Corbally M. Cholecystectomy versus cholecystolithotomy for cholelithiasis in childhood: long-term outcome. J Pediatr Surg. (2001) 36(10):1518–21. 10.1053/jpsu.2001.27035 11584400

[16] Zha Y Zhou ZZ Chen XR Gan P Tan J. Gallbladder-preserving cholelithotomy in laparoscopic and flexible choledochoscopic era: a report of 316 cases. Surg Laparosc Endosc Percutan Tech. (2013) 23(2):167–70. 10.1097/SLE.0b013e31828a0b5f 23579512

[17] Liu Y Jin S Li Y Xu R Pang W Wang K Treatment of asymptomatic gallstones in children with hereditary spherocytosis requiring splenectomy. J Pediatr Surg. (2023) 58(4):756–61. 10.1016/j.jpedsurg.2022.11.012 36588038

